# Surgery versus stereotactic radiotherapy for treatment of pulmonary metastases. A systematic review of literature

**DOI:** 10.2144/fsoa-2019-0120

**Published:** 2020-04-15

**Authors:** Francesco Londero, William Grossi, Angelo Morelli, Orlando Parise, Gianluca Masullo, Cecilia Tetta, Ugolino Livi, Jos G Maessen, Sandro Gelsomino

**Affiliations:** 1 Department of Cardiothoracic Surgery, S. Maria della Misericordia University Hospital, Udine, Italy; 2 Cardiovascular Research Institute Maastricht, Maastricht, The Netherlands; 3 Radiology Department, Rizzoli Institute, Bologna, Italy

**Keywords:** lung, oligorecurrence, oligometastases, radiotherapy, surgery

## Abstract

It is not clear as to which is the best treatment among surgery and stereotactic radiotherapy (SBRT) for lung oligometastases. A systematic review of literature with *a priori* selection criteria was conducted on articles on the treatment of pulmonary metastases with surgery or SBRT. Only original articles with a population of patients of more than 50 were selected. After final selection, 61 articles on surgical treatment and 18 on SBRT were included. No difference was encountered in short-term survival between pulmonary metastasectomy and SBRT. In the long-term surgery seems to guarantee better survival rates. Mortality and morbidity after treatment are 0–4.7% and 0–23% for surgery, and 0–2% and 4–31% for SBRT. Surgical metastasectomy remains the treatment of choice for pulmonary oligometastases.

## Lay abstract

Patients with metastatic cancer with a limited number of deposits may benefit from surgical removal or irradiation of tumor nodules in addiction to chemotherapy. Surgical resection has been demonstrated to improve survival and, in some cases, can be curative. Stereotactic radiotherapy is emerging as a less invasive alternative to surgery, but settings and implications of the two treatments are profoundly different. The two techniques show similar results in the short-term, with lower complications rates for radiotherapy, while in the long-term surgery seems to guarantee higher survival rates.

Standard treatment for metastatic disease has historically been systemic chemotherapy, usually with only limited benefit on survival [1]. Evidence drawn from retrospective series have documented how patients with a limited pattern of disease demonstrate improved long-term outcomes when submitted to local aggressive treatments [2]. While complete surgical excision has always been acknowledged as the upfront treatment for this condition [3], in more recent years other techniques have been introduced, such as stereotactic radiotherapy (SBRT), which is a hypofractionated, high-dose delivery of radiation on a target tumor. This seems to guarantee comparable results with surgery in many retrospective series in terms of control of disease [4–6], with the advantage of lower invasiveness and postprocedural complications. To our knowledge, there is no prospective trial or systematic review to date comparing the effectiveness and safety of the two treatment modalities.

Therefore, we reviewed both retrospective and prospective case series, which assessed the efficacy of pulmonary metastasectomy or SBRT in patients with an oligometastatic state in terms of prognostic outcomes, control of disease and safety of treatment.

## Materials & methods

### Search strategy

A literature search was conducted in accordance with the Preferred Reporting Items for Systematic Review and Metanalyses (PRISMA) [7]. An unrestricted literature search was performed using PubMed, EMBASE, Web of Science and Google Scholar Databases, as well as congress proceedings from major Thoracic and Cardiothoracic society meetings.

Search terms were: ‘lung metastases’ ‘surgery’ OR ‘stereotactic radiotherapy.’ The search strategy was decided by two authors (FL and WG) and approved by another reviewer (SG).

The literature was limited to articles published in English. References of original articles were reviewed manually and cross-checked for other relevant reports.

### Selection criteria & quality assessment

The literature research was conducted by previously defining a searching strategy using the PICOS method [8]. Initial selection criteria for study inclusion were:

- Type of study: original articles, no review or safety/feasibility studies;
- Primary tumor: no restrictions;
- Type of metastases: only pulmonary;
- Treatment modality: surgery or SBRT;
- Population >50;
- Adequate information regarding prognostic results, adverse events or both.

Since the first description of the oligometastatic state dates back to 1995, we included only articles published since 1995. Last search was run on 15th January 2018.

Two authors (FL and WG) selected the study for inclusion, extracted studies, as well as patient information and outcomes. Two reviewers (JGM and UL) independently assessed eligibility of the studies and risk of bias.

Studies assessing the efficacy of radiofrequency ablation were excluded since we decided to concentrate on the comparison between surgery and SBRT. If case sample size and/or treatment results have not been clearly reported in the abstract, articles were assessed via full text analysis for final inclusion in the review. In case of disagreement, consensus between reviewers was obtained through conjunct review and discussion throughout all the selection process.

We tried to contact one author to gather some information regarding results reported in his article [9], but we did not succeed in doing so.

Data were placed into a collection sheet and information included: characteristics of populations of patients (median age, sex ratio), inclusion/exclusion criteria of patients in the relative studies and basic disease (primary tumor, number of pulmonary lesions, diameter of lung deposits); interventions (surgery, SBRT or both) and treatment modalities (for surgery: surgical approach, kind of resection and postresectional status; for SBRT: radiation doses, number of fractions of treatment delivery); length of follow-up; outcomes of treatments (survival, rates of recurrence, progression-free survival [PFS], local control [LC], mortality, adverse events and surgical complications).

Articles were reviewed for risk of reporting biases or evident errors. Since both treatment modalities deal with a neoplastic condition the main outcome of interest was survival and incidence of recurrence. Where available we also collected data on the tolerability and safety of both treatments, in the sense of incidence of adverse events, complications and mortality within the first 30 days.

Given the wide heterogeneity of reporting clinical information across the different studies, we processed some data in order to get them aligned and comparable: gender prevalence has been modified into male/female number; age has been reported as it was originally as median and interquartile range, median and overall range or mean ± standard deviation; primary tumor histology, type of treatment (approach, kind of resection and postresection status for surgery and dose of radiation and number of frames for SBRT), outcomes, morbidity, mortality and complications have been translated, where necessary, into percentages instead of absolute values.

## Results

### Study selection

**Figure 1.**
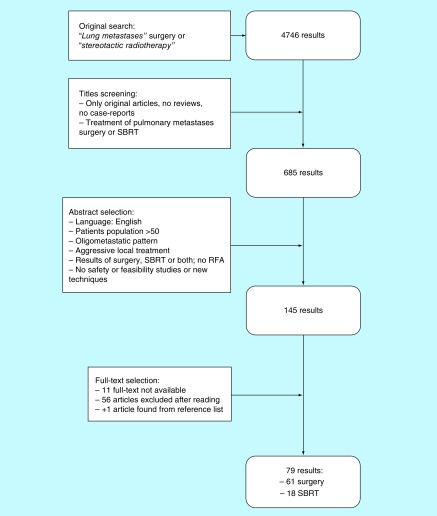
Flowchart of articles selection process. RFA: Radiofrequency ablation; SBRT: Surgery and stereotactic radiotherapy.

Article selection process is reported in the diagram in Figure 1. The first search on electronic databases, after exclusion of duplicates, led to 4746 results. The first screening led to 685 abstracts, which were further scrutinized: 145 original works were selected based on language (only English), sample size (>50 patients), pattern of disease (oligometastatic) and treatment performed with curative intent (SBRT and surgery, no radiofrequency ablation, cryoablation or other local treatments); safety or feasibility studies on new techniques were also excluded. If a manuscript reported on comparative results between surgical metastasectomy and SBRT, but only one of the two populations of study was greater than 50 patients, data were collected only on the arm of treatment meeting the minimal requirements on sample size. Last selection step was performed on full text analysis. One study was identified after checking references lists and included in the review. After final selection, a total of 79 studies were included and: of these, 61 were reports on surgical case series and 18 reported on the results of SBRT.

### Bias

The majority of surgical studies (50.8%) do not report the size of resected lesions, which is a well-known prognostic factor and an important information regarding the burden of disease. Moreover, a few included articles reported the number of metastases ranging over five, which is the original uplimit for the definition of an oligometastatic state. Conversely, a minority of studies reported information regarding a history of previous local ablative treatments for oligometastases or the synchronous/metachronous development of metastases. Considering the relevant impact of these aspects on the patients’ prognosis [10], this undoubtedly represents a huge limitation of the analysis. We observed how a relevant proportion of publications on surgical series report percentages of incomplete resections ranging between 3 and 21.3%: this represents evidence of involved margins of resection in most cases, but some studies reported percentages of macroscopic residual disease. Including this subset of patients in survival analysis may have a considerable impact on survival rates of the whole population, considering the known detrimental effect of incomplete resection on prognosis [11].

None of the included studies on SBRT reported that a histological confirmation of the metastatic nature of nodules was carried out before or after treatment. This might suggest that a proportion of the irradiated nodules were not neoplastic, which has potential impact on the survival and recurrence analyses.

Although a statistical analysis on the difference of populations’ age between surgical and SBRT series could not be performed, median/mean age tend to be higher in SBRT studies. This point should be considered when comparing results of both treatments, since populations might be different in terms of baseline conditions, with relevant effects on outcomes.

### Patient population & tumor characteristics

#### Surgery

Results concerning study populations and the characteristics of tumors in surgical series are reported in Table 1. Among the 68 manuscripts on surgical series, only one was a prospective observational study, whereas the others were all retrospective. Some works report how their data were derived from prospectively maintained databases, but none of them clearly reported a prospective protocol for their study.

**Table 1.** Surgery: study populations’ and tumors’ characteristics.

Study (year)	Type	Pat	Age (range)	M:F	Primary tumor	Lesions (n)	Tumor diameter	Ref.
Fourquier *et al.* (1997)	R	50	59^‡^ (40–78)	38:12	RCC	134	ns	[12]
Robert *et al.* (1997)	R	276	38^‡^ (5–78)	193:83	S: 46%, T: 32%, C: 19%, M: 1.8%, O: 1.4%	1: 42.7%; >1: 57.3%	ns	[44]
Pastorino *et al.* (1997)	R	5206	46^‡^ (2–93)	2932:2273	E: 43%, S: 42%, GCC: 7%, M: 6%, O: 2%	1: 45.8%; >1: 54.2%	ns	[10]
van der Veen *et al.* (1998)	R	78	51^‡^ (17– 73)	46:32	A: 26.9%, S: 24.4%, Te: 19.2%, RCC: 16.7%, M: 12.8%	ns	ns	[13]
Abecasis *et al.* (1999)	R	85	48.5^‡^ (16–79)	41:44	STS: 21.2%, Os: 15.3%, HN: 14.1%, B: 11.8%, CRC: 8.2%, CC: 5.9%, M: 4.7%, RCC: 3.5%, Bl: 3.5%, O: 11.8%	1: 74.1%; >1: 25.9%	ns	[45]
Kolodziejski *et al.* (1999)	R	93	50^†^ (17–71)	49:44	RCC: 25%, CRC: 11%, STS: 11%, M: 10%, B: 10%, U: 7%, NSCLC: 6%, Te: 4%, O: 16%	1: 86%; 2: 8,6%; 3: 5.4%	ns	[35]
Leo *et al.* (2000)	R	282	47^†^	156:126	M	1: 65.1%; >1: 34.9%	ns	[46]
Friedel *et al.* (2001)	R	467	53^‡^ (21–87)	All F	B	1: 65.9%; >1: 44.1%	ns	[47]
Piltz *et al.* (2002)	R	105	59^‡^ (35–78)	73:32	RCC	1: 46.7%; >1 53.3%	ns	[48]
Planchard *et al.* (2003)	R	125	53^‡^ (30–82)	All F	B	1: 59.2%; >1: 40.8%	20.5^†^ (5–70)	[49]
Monteiro *et al.* (2004)	R	78	59^‡^ (25–78)	41:37	Co: 24.4%, Re: 20.5%, B: 15.4%, U: 7.7%, HN: 6.4%, UT: 7.7%, Te: 3.8%, O: 14.1%	1: 67.9%; >1: 32.1%	<30: 64.1%; >30: 35.9%	[36]
Vogelsang *et al.* (2004)	R	75	58^‡^ (33–82)	42:33	CRC	1: 65.6%; >1: 34.4%	20^‡^ (5–80)	[50]
Shiono *et al.* (2005)	R	87	61^‡^ (23–82)	57:30	CRC	1: 75.9%; >1: 24.1%	18^‡^ (7–6.7)	[51]
Casali *et al.* (2006)	R	142	60.9 (33–89)	74:68	CRC: 44%, RCC: 22%, B: 16%, HN: 5%, U: 4%, SG: 3%, Bl: 2%, O: 4%	1: 65%; >1: 35%	ns	[52]
Furak *et al.* (2006)	R	52	60.5^†^ (20–86)	32:20	CRC: 37%, RCC 19%, M: 15%, B: 6%, Bl: 6%, U: 4%, Os: 4%, Te: 2%, SG: 2%, O: 2%, Th: 2%, L: 2%	1: 82.7%; >1: 17.3%	ns	[53]
Harting *et al.* (2006)	R	93	<10: 17.2%; 10–21: 82.8%	ns	S	1: 28.3%; >1: 71.7%	ns	[54]
Rena *et al.* (2006)	R	202	64^‡^ (24–80)	107:95	CRC: 46.4%, RCC: 15.2% B: 11.4%, Bl: 10.2%, HN: 8.2%, U: 4.3%, O: 4.3%	1: 72.7%; >1: 27.3%	ns	[55]
Assouad *et al.* (2007)	R	65	62.1^†^ (42–82)	52:13	RCC	ns	ns	[15]
Melloni *et al.* (2007)	R	81	61^‡^ (38–83)	49:32	CRC	1: 54.3%; >1: 45.7%	20^‡^ (10–130)	[16]
Veronesi *et al.* (2007)	R	124	59^‡^ (24–82)	61:63	C: 43.5%, S: 12.1%, HN: 6.4%, UT: 12.9%, B: 8.9%, Gy: 6.4%, M: 4.8%, GCC: 2.4%, O: 2.4%	1: 58.9%; >1 41.1%	ns	[56]
Dahabre *et al.* (2008)	R	57	60^‡^ (30–87)	34:23	CRC	1: 59.65%; >1: 40.35%	ns	[9]
Smith *et al.* (2008)	R	94	49^‡^ (9–75)	47:47	S	1: 36.2%; >1: 63.8%	ns	[17]
Yoshimoto *et al.* (2008)	R	90	55.1^†^ (32–77)	All F	B	1: 86.7%; >1: 13.3%	23^†^ (8–48)	[37]
Winter *et al.* (2008)	R	67	57.1^†^	53:14	HN	1: 79.1%; >1: 20.9%	<30: 79.1%; >30: 20.9%	[57]
Lin *et al.* (2009)	R	63	58.7^†^ (32–78)	39:24	CRC	1: 65%; >1: 35%	27.7^†^ (5–86)	[58]
Rama *et al.* (2009)	R	61	61.2^†^ (30–80)	42:19	CRC	1: 60.6%; >1: 39.4%	>30: 72%; >30: 28%	[59]
Takakura *et al.* (2009)	R	56	64^‡^ (42–76)	26:30	CRC	1: 68.5%; >1: 31.5%	20^‡^ (5–88)	[60]
Borasio *et al.* (2010)	R	137	63.8^†^ (35–82)	82:55	CRC	1: 59.9%; >1: 40.1%	20^‡^ (2.5–70.0)	[61]
Riquet *et al.* (2010)	R	127	65^†^ (36–85)	74:53	CRC	1: 59.0%; >1: 41.0%	30.2^†^ (2–90)	[62]
Sardenberg *et al.* (2010)	R	77	<45: 48.1%; >45: 51.9%	37:40	S	1: 55.9%; >1: 44.1%	<20: 27.4%; >20: 72.6%	[63]
Tacconi *et al.* (2010)	R	87	60.5^†^ (16–82)	47:40	CRC: 51.7%, RCC: 14.9%, U: 8%, L: 5.7%, B: 3.4%, Pa: 2.3%, Ov: 2.3%, S: 8%, M: 3.4%	191	ns	[64]
Casiraghi *et al.* (2011)	R	575	58^†^ (14–83)	247:328	E: 75.8%, GI: 36.5%, B: 8.9%, UT: 14.9%, Gy: 7.3%, HN: 7.5%, Ty: 0.7%, S: 16.3%, M: 5.7%, GCC: 2.1%	1: 36.3%; >1: 63.7%	ns	[65]
Long *et al.* (2011)	R	55	47^‡^ (12–88)	40:15	CRC: 25.4%, HCC: 16.4%, RCC: 10.8%, Os: 5.5%, NSCLC: 5.5%, HN: 5.5%, U: 3.6%, O: 27.2%	174	ns	[66]
Stephens *et al.* (2011)	R	81	43.5^‡^	45:36	S	mean 5.5/patient	ns	[67]
Zabaleta *et al.* (2011)	R	84	65^‡^ (40–82)	60:24	CRC	1: 77.4%; >1: 22.6%	<20: 42.9%; >20: 57.1%	[68]
Blackmon *et al.* (2012)	R	229	60^‡^ (24–82)	129:100	CRC	1: 76.9%; >1: 23.1%	15^‡^ (2–145)	[18]
Chua *et al.* (2012)	R	292	59^‡^ (19–84)	208:84	M	1: 59.2%; >1: 40.8%	20^‡^ (10–150)	[69]
Goonerante *et al.* (2012)	R	106	64^‡^ (IQR: 16–46)	59:57	CRC	1: 75.5%; >1: 24.5%	20^‡^ (14–38)	[19]
Hirosawa *et al.* (2012)	R	266	64^‡^ (25–86)	168:98	CRC	1: 45.1%; >1: 54.9%	18^‡^ (4–70)	[70]
Mizuno *et al.* (2012)	R	52	41^‡^ (7–74)	34:18	S	1: 53.8%; >1: 46.2%	ns	[20]
Lo Faso *et al.* (2013)	R	164	64.17^†^ ± 14.4	100:64	S: 17.1%, M: 4.3%, GCC: 1.8%, E: 76.38%	1: 59.1%; >1: 40.9%	ns	[71]
Poletti *et al.* (2013)	R	119	52^‡^ (15–75)	68:51	CRC: 47.9%, S: 21.8%, HN: 7.5%, Go: 5.9%	511	ns	[38]
Cho *et al.* (2014)	R	626	62^‡^ (23–86)	390:136	CRC	1^‡^ (1–21)	16	[72]
Renaud *et al.* (2014)	R	320	63^‡^ (27–86)	215:105	CRC	1: 49.4%; >1: 50.6%	ns	[73]
Renaud *et al.* (2014)	R	122	63.3^†^ (43–82)	84:38	RCC	1: 35.2%; >1: 64.8%	ns	[74]
Zampino *et al.* (2014)	R	199	61^‡^ (19–82)	125:74	CRC	1: 47.7%; >1: 52.3%	ns	[75]
Booth *et al.* (2015)	R	709	65^†^	424:285	CRC	ns	ns	[41]
Hou *et al.* (2015)	R	114	62^‡^ (45–76)	74:40	CRC	151	40^‡^ (10–70)	[21]
Seebacher *et al.* (2015)	R	209	62.4^†^ (30–81)	110:99	CRC: 40.2%, RCC: 18.2%, B: 10.5%, U: 6.7%, HN: 4.8%, M: 4.3%, S: 3.8%, Th: 3.8%, UT: 3.3%, O: 4.8%	3.4^†^ ± 4.1 per pat	26^†^	[76]
Guerrera *et al.* (2016)	R	188	66^‡^ (IQR: 58–72)	109:79	CRC	1: 53%; >1: 47%	30^‡^	[77]
Filippi *et al.* (2016)	R	142	66^‡^ (IQR: 59–72)	87:55	CRC	1: 54.9%; >1: 45.1%	15^‡^	[30]
Franzke *et al.* (2016)	R	178	59.3^†^ (17–85.4)	110:68	CRC: 30.3%, RCC: 16.9%, S: 14.6%, M: 10.6%, GCC: 3.9%, B: 3.4%, O: 20.3%	1: 55.6%; >1: 44.4%	19.6^†^ (3–120)	[78]
Girelli *et al.* (2016)	R	109	48^‡^ (IQR: 31–65)	58:51	SG	1: 26.6%; >1: 73.4%	ns	[79]
Okiror *et al.* (2016)	R	66	51^‡^ (16–79)	39:27	S	3^‡^ (1–9)	ns	[80]
Guerrini *et al.* (2016)	R	224	64^†^ ± 14.4	140:84	S: 12.5%, M: 3%, GCC: 1.3%, CRC: 59.8%, B: 10.7%, UT: 5.8%, Gy: 0.9%, HN: 5.8%	1: 56.7% >1: 43.3%	ns	[39]
Gafencu *et al.* (2017)	R	327	50.57^†^ ± 16.21	166:161	S	1–3: 86.5%; 4+: 23.5	21.7^†^	[22]
Kim *et al.* (2017)	R	129	56^‡^ (33–76)	77:52	CRC	1: 78.3%; >1: 21.7%	<10: 33.3%; >10: 66.7%	[23]
Shiomi *et al.* (2017)	R	100	64.7^†^ ± 10.0	56:44	CRC	1: 72%; >1: 28%	<20: 68%; >20: 32%	[81]
Sun *et al.* (2017)	R	154	<65: 71.4%; >65: 28.6%	101:53	CRC	1: 81.2%; >1: 18.8%	21^‡^	[82]
Shiono *et al.* (2017)	R	553	66^‡^ (29–89)	314:139	CRC	Median per pat: 1 (1–8)	15^‡^ (5–51)	[14]
Lodeweges *et al.* (2017)	R	68	61^‡^ (18–80)	37:31	CRC: 57.3%, S: 26.5%, RCC: 7.3%, O: 8.8%	109 (1.60 per pat)	20^†^ (1.7–2.4)	[24]

Values expressed as median/mean (range):

† Mean.

‡ Median.

A: Adenocarcinoma; B: Breast; Bl: Bladder; C: Carcinoma; CC: Cervical carcinoma; Co: Colon; CRC: Colorectal cancer; E: Epithelial; En: Endometrial; GCC: Germ-cell cancer; GI: Gastrointestinal tract; Go: Gonads; Gy: Gynecological tract; HN: Head & neck; IQR: Interquartile range; L: Larynx; M: Melanoma; M/F: Male/female; ns: Not stated; NSCLC: Non-small-cell lung cancer; O: Other; Os: Osteosarcoma; Ov: Ovary; P: Prospective; Pa: Prostate; Pat: Number of patients; R: Retrospective; RCC: Renal cell carcinoma; Re: Rectum; S: Sarcoma; SG: Salivary gland; STS: Soft tissue sarcoma; T: Teratoma; Te: Testicle; Th: Thyroid; Ty: Thymus; U: Uterine; UL: Uterine leyomiosarcoma; UT: Urinary tract; VATS: Video-assisted thoracic surgery.

Population size ranged between 50 and 5206, with a median sample size of 114 patients (interquartile range [IQR]: 78–202). Apart from few articles reporting outcomes on the treatment of patients with metastases from sarcoma – where subjects were usually younger – mean or median values ranged uniformly between 48 and 66 years.

With the obvious exception of the series of patients treated for metastases from breast cancer, the majority of works reported a marked prevalence of males in their populations. The larger proportion of studies pertained to populations of patients with metastases from colorectal cancer only (24 articles) or various primary tumor origin (19 articles). Seven articles illustrated results of patients treated for metastases from primary sarcoma. The large majority of articles (95.1%) report number of metastases, but the way this information is detailed vary quite extensively across studies: number of pulmonary deposits is referred as mean/patient, total number, single versus multiple or stratified per number with relative percentages/absolute numbers. The same observation can be made regarding size of pulmonary metastases, but in this case the information is provided in less than half of the manuscripts. A quite high conformity has been found in reported inclusion criteria. However, in 14 articles (20.6%), no reference was made to criteria for enrolling patients in the studies [12–25].

#### SBRT

Information regarding the study population and tumor characteristics of SBRT series are reported in Table 2. With the exception of one prospective study, all included papers regarding the results of SBRT were retrospective case series. Median sample population was 66 patients (IQR: 61–96, range: 50–700). Age has usually been reported as median or mean and range, whereas in three articles it has been stratified as 60 years the cutoff for subdivision. Only in one article median age was <60 years [26], whereas most populations' median/mean age ranged between 65 and 71. Male sex was prevalent in the majority of articles (88.9%) with a male/female ratio ranging between 0.93 and 6.12. Regarding tumor characteristics, 77.8% of articles included patients with pulmonary metastases arising from various primary tumors. Three articles (16.7%) were focused on results of irradiated metastases from colorectal cancer and only one on the recurrence of lung tumors. The number of lung deposits has been presented as absolute numbers, mean/patient or stratified with relative number/percentages. Tumor size has been variously described as diameter or volume, this latter both as gross tumor volume (GTV) or planning tumor volume (PTV). In 22.2% of papers, tumor size has been reported as stratified in classes, in a quite heterogeneous way across studies. Patients’ inclusion criteria were reported in 83.3% of articles, 72.2% also described definition criteria for an oligometastatic state and, in most of the publications, the maximum size of metastases accepted for irradiation <50 mm.

**Table 2.** Stereotactic radiotherapy: studies’ population and tumor characteristics.

Study (year)	Type	Pat	Age (range)	M:F	Primary tumor	Lesions (n)	Tumor diameter	Ref.
Hof *et al.* (2007)	R	61	65.9^†^ (25–87)	40:21	NSCLC: 50.8%; CRC: 13.1%; B: 6.5%; O: 29.5%	71	GTV 10 cm^3^^†^ (1–53)	[6]
Ricardi *et al.* (2011)	R	61	70^†^ (46–86)	2.34:1	NSCLC: 53.5%; CRC: 21.3%; Pa: 3.3%; HCC: 3.3%; HN: 3.3%; O: 12.8%	77	20 mm^†^ (7–45)	[83]
Zhang *et al.* (2011)	R	71	59^†^ (15–84)	45:26	NSCLC: 18.3%; CRC: 15.5%; HN: 14.1%; S: 11.3%; HCC: 11.3%; RCC: 8.5%; B: 7.0%; O: 14.1%	172	21 mm^†^ (0.9–7.9)	[25]
Oh *et al.* (2012)	R	57	<60: 28%; >60: 72%	49:8	NSCLC: 49.2%; HCC: 13.4%; CRC: 10.5%; HN: 16.4%; O: 10.5%	67	<25 mm: 86.6%; >25 mm: 13.4%	[34]
Filippi *et al.* (2014)	R	67	71^†^ (40–86)	44:31	NSCLC: 37.4%; CRC: 40.3%; M: 7.5%; HN: 4.5%; HCC: 2.9%; Oe: 2.9%; B: 1.5%; RCC: 1.5%; Pr: 1.5%	90	17 mm^†^ (7–38)	[5]
Osti *et al.* (2013)	P	66	68^†^ (25–89)	32:34	NSCLC: 18%; CRC: 35%; B: 17%; O: 30%	103	<10 cc: 62%; >10 cc: 38%	[29]
Siva *et al.* (2015)	R	65	69^†^ (IQR: 61–78)	38:27	CRC: 31%; NSCLC: 25%; HN: 11%; STS: 8%; O: 25%	1: 78.5%; >1: 21.5%	ns	[84]
Aoki *et al.* (2015)	R	66	71^†^ (27–87)	44:22	NSCLC: 47%; CRC: 19.7%; HN: 15.1%; Oe: 4.5%; U: 4.5%; O: 9.1%	76	≤ 30: 92.1%; >30: 7.9%	[85]
Binkley *et al.* (2015)	R	77	60^†^ (30–93)	40:37	NSCLC: 17.2%; CRC: 21.3%; S: 15.6%; O: 45.9%	122	GTV: 3.7 ml^†^ (0.2–61.4)	[86]
Jung *et al.* (2015)	R	50	65^†^ (30–82)	ns	CRC	79	GTV: 1.5 ml^†^ (0.2–34.8)	[87]
De Rose *et al.* (2016)	R	60	70.5^†^ (33–89)	40:20	NSCLC	90	Cumulative volume: 17.5 cm^3^^‡^ (0.9–184)	[88]
Kinj *et al.* (2016)	R	53	69^†^ (47–84)	35:18	Re: 32.1%; Co: 67.9%	87	16 mm^†^ (3–70)	[33]
Helou *et al.* (2016)	R	120	67^‡^ ± 11 yrs	58:62	NSCLC: 31.3%; RCC: 25.3%; B: 21.7%; O: 21.7%	184	15 mm^†^ (IQR: 10–25)	[89]
Rieber *et al.* (2016)	R	700	67^†^ (6.4–99.9)	449:251	NSCLC: 30%; CRC: 21.9%; S: 7.3%; RCC: 6.9%; B: 6.2%; M: 3.4%; Oe: 2.6%; O: 21.7%	1: 42.4%; >1: 57.6%	22 mm^†^ (4–94)	[4]
Yamashita *et al.* (2016)	R	96	72^†^ (25–88)	65:31	CRC: 26%; NSCLC: 25%; HN: 8%; U: 8%; O: 32%	1: 79.2%; >1: 20.8%	19 mm^†^ (0.6–4.2)	[90]
Franceschini *et al.* (2017)	R	200	69^†^ (22–90)	123:77	RCC: 12%; M: 4.5%; HCC: 10%; SG: 3.5%; S: 20.5%; CRC: 49.5%	1: 64%; >1: 36%	ns	[91]
Ricco *et al.* (2017)	R	447	69^†^ (18–93)	223:221	B: 9.2%; CRC: 25.7%; HN: 11.4%; NSCLC: 16.6%; RCC: 8.1%; M: 6.5%; O: 22.1%	1^†^ (1–3)	10.58 cc^†^ (0.1–654.5)	[92]
Qiu *et al.* (2018)	R	65	>60: 60%; <60: 40%	37:28	CRC	1: 36.9%; >1: 63.1%	<10 mm: 27.7%; >1: 72.3%	[93]

Values expressed as median/mean (range):

† Median.

‡ Mean.

B: Breast; Co: Colon; CRC: Colorectal Cancer; GTV: Gross tumor volume; HCC: Hepatocarcinoma; HN: Head & neck; IQR: Interquartile range; M: Melanoma; ns: Not stated; NSCLC: Non-small-cell lung cancer; O: Other; Oe: Oesophagus; P: Prospective; Pa: Pancreas; Pat: Patient; Pr: Prostate; R: Retrospective; RCC: Renal Cell Carcinoma; Re: Rectal; S: Sarcoma; SG: Salivary gland; STS: Soft tissue sarcoma; U: Uterine.

### Treatment

#### Surgery

The description of surgical approaches and length of follow-up are presented in Table 3. A common attitude was determined regarding surgical approach and extent of resection. The most common surgical approach reported was thoracotomy. Interestingly, a positive trend has been found in more recent publications in the reported percentages of resection performed via thoracoscopy (video-assisted thoracic surgery [VATS]). Bilateral localizations of disease have been treated with different approaches: staged thoracotomies within a short interval of time represent the main proportion, while other approaches such as median sternotomy, synchronous bilateral thoracotomy, hand-assisted procedures and clamshell incision have been described in several papers. A large proportion of authors favored a lung-sparing resection, atypical resection, which was also the most frequent intervention performed. Wedge resections were reported as almost the totality of procedure performed in one paper employing a substernal hand-assisted approach, whereas they represent only 11% of resections performed in a series of patients with metastases from breast cancer. Apart from these cases, in many publications atypical resections have been employed in approximately 60–70% of surgical procedures. Extensive resections represent a minor proportion in the majority of works, but pneumonectomies constitute up to 19.5% of resections performed in some series. Several studies mention percentages of patients were residual disease could be assumed (microscopic, R1) or evident (macroscopic, R2) after metastasectomy. A R0 resection was achieved in more than 90% of cases in most articles, but rates of incomplete resection and even macroscopic residual disease could reach 20% in several reports [18].

**Table 3.** Surgery: treatment and follow-up.

Study (year)	Resection	Approach	R0	Follow-up (mo)	Ref.
Fourquier *et al.* (1997)	W: 56%; S: 6%; L: 34%; SL: 2%; P: 10%	Th: 64%; S: 12%; BTh: 8%; STh: 14%	ns	42^†^ (1–200)	[12]
Robert *et al.* (1997)	W: 66%; S: 5%; L: 24%; P: 5%	Th: 68.8%; TL: 5.1%; St: 17.4%; STh: 8.7%	90%	38^‡^ (0–161)	[44]
Pastorino *et al.* (1997)	W: 66.7%; S: 8.6%; L: 21.3%; P: 2.5%	Th: 59.7%; BTh: 11%; St: 27.2%; V: 1.7%	88%	46^†^	[10]
van der Veen *et al.* (1998)	W: 94.9%; L: 5.1%	St	82.1%	22^‡^ (1–132)	[13]
Abecasis *et al.* (1999)	W: 55.5%; S: 2.0%; L: 32.9%; B: 7.6%; P: 2.0%	Th: 91.5%; STh: 6.3%; Cl: 1.1%; V: 1.1%	ns	22^‡^ (1–146)	[45]
Kolodziejski *et al.* (1999)	W: 33%; S: 13%; L: 28%; P: 19.5%; ETh: 6.5%	Th: 99%; St: 1%	ns	ns	[35]
Leo *et al.* (2000)	P: 4.5%; L: 26.8%; W/S: 68.7%	U: 79.4%; Bi: 20.6%	ns	ns	[46]
Friedel *et al.* (2001)	W: 61.2%; S: 12.0%; L: 24.4%; P: 1.3%; ns: 1.1%	Th: 75.4%; St: 20.4%; V: 3.6%; ns: 0.6%	84%	34^†^ (0–240)	[47]
Piltz *et al.* (2002)	W: 78.6%; S: 6%; L: 12.6%; B: 3.8%	ns	ns	ns	[48]
Planchard *et al.* (2003)	W/S: 74%; L: 20%; C: 6%	Th: 92%; BTh: 8%	ns	112.8^†^ (2–264)	[49]
Monteiro *et al.* (2004)	W: 70.4%; L: 28.6%; P: 1%	ns	ns	40.8^†^ (3–162)	[36]
Vogelsang *et al.* (2004)	An: 29.8%; W: 70.2%	ns	100%	33^‡^ (4–116)	[50]
Shiono *et al.* (2005)	W: 49.5%; S: 19.5%; L: 28.8%; B: 1.1%; P: 1.1%	ns	ns	32^‡^ (1–110)	[51]
Casali *et al.* (2006)	W: 69%; L: 28%; B: 3%	ns	Ns	ns	[52]
Furak *et al.* (2006)	W: 54%; L: 42%; P: 4%	U: 90%; Bi: 10%	ns	ns	[53]
Harting *et al.* (2006)	ns	U: 58.6%; Bi: 41.4%	64.7%	34.8^‡^ (9–241)	[54]
Rena *et al.* (2006)	W: 62.5%; S: 5.6%; L: 23.8%; C: 4.5%; B: 1.6%; P: 1.9%	Th: 95.5%; St: 1.5%; Cl: 0.5%; STh: 2.5%	83.6%	25^‡^ (1–218)	[55]
Assouad *et al.* (2007)	W: 56.6%; L: 20.5%; S: 13.2%; C: 6.0%; P: 3.6%	U: 79.1%; Bi: 20.9%	ns	ns	[15]
Melloni *et al.* (2007)	W: 69.3%; L: 29.5%; P: 1.2%	Th: 93.2%; St/STh: 6.8%	ns	20^‡^ (4–154)	[16]
Veronesi *et al.* (2007)	W: 40.3%; S: 21.6%; L: 35.2%; P: 2.9%	Th	ns	31^†^ (2–82)	[56]
Dahabre *et al.* (2008)	W/S: 45%; L: 41%; B 7%; P: 7%	ns	91.2%	ns	[9]
Smith *et al.* (2008)	W: 78.7%; L: 18.1%; P: 3.2%	ns	78.7%	Minimum 60	[17]
Yoshimoto *et al.* (2008)	W: 11.1%; S: 12.2%; L: 75.6%; P: 1.1%	ns	ns	79.2^‡^	[37]
Winter *et al.* (2008)	W: 77.8%; S: 12.3%; L: 6.2%; P: 1.2%; B: 2.5%	ns	ns	ns	[57]
Lin *et al.* (2009)	W: 58.7%; L: 41.3%	ns	ns	37.3^‡^ (12–122)	[58]
Rama *et al.* (2009)	W: 78%; L: 17%; O: 5%	Th: 84%; BTh: 15%; St: 1%	ns	ns	[59]
Takakura *et al.* (2009)	W/S: 50%; L: 50%	ns	ns	30^‡^ (4–126)	[60]
Borasio *et al.* (2010)	W: 70.8%; S: 9.5%; L: 19%; B: 0.7%	Th: 85.4%; St: 11.7%; BTh: 0.7%; V: 2.2%	ns	36.2^‡^ (4.6–126)	[61]
Riquet *et al. 2010*	W/S: 56%; L: 36.3%; P: 7.7%	ns	ns	46^‡^ (2–256)	[62]
Sardenberg *et al. 2010*	W/S: 81.8%; L: 15.6%; Bio: 2.6%	ns	ns	36.7^‡^ (10–138)	[63]
Tacconi *et al.* (2010)	W	HAV	ns	65^†^ (8–72)	[64]
Casiraghi *et al.* (2011)	Tu: 20.8%; W: 39.2%; S:11.8%; L/B: 27%; P: 1.2%	Th: 68.8%; STh: 26.3%; St: 2%; HCl: 0.2%; O: 0.4%	85.2%	34^†^	[65]
Long *et al.* (2011)	W: 99.5%; L: 0.5%	HAV	ns	27.4 (1–74)	[66]
Stephens *et al.* (2011)	W/S: 85%; L/P: 15%	ns	ns	27.0^†^	[67]
Zabaleta *et al.* (2011)	W: 47.5%; L: 37.6%; B: 1.0%; P: 5.0%; C: 5.0%	Th: 91.2%; V: 6.5%; O: 2.0%	ns	43^‡^ (0–130)	[68]
Blackmon *et al.* (2012)	W: 72.5%; S: 12.2%; L: 15.3%	V: 16.2%; St/Cl: 8.7%; Th: 73.4%; O: 1.7%	ns	37.2^‡^	[18]
Chua *et al.* (2012)	W/S: 51%; O: 49%	U: 94%; Bi: 6%	80%	20^‡^ (0–255)	[69]
Goonerante *et al.* (2012)	W: 54.1%; L: 42.4%; C: 7.4%; P: 2.1%	ns	ns	30^‡^ (IQR: 16–46)	[19]
Hirosawa *et al.* (2012)	W: 42.3%; S: 38.3%; L: 32.4%	U: 87.6%; Bi: 22.4%	100%	74^‡^ (1–188)	[70]
Mizuno *et al.* (2012)	W: 84%; S: 9%; L: 7%	V: 59%; Th: 41%	92%	ns	[20]
Lo Faso *et al.* (2013)	W: 64.1%; S: 10.4%; L: 25%; P: 0.5%	V	97%	38^†^	[71]
Poletti *et al.* (2013)	W/S: 51.3%; Tu: 29.9%; L/B: 15.6%; P: 3.2%	ns	ns	ns	[38]
Cho *et al.* (2014)	W: 77.3%; S: 5.9%; L: 15.6%; P: 0.8%	Th/St: 76.1%; V: 23.8%	ns	45.5^‡^ (23.0–94.2)	[72]
Renaud *et al.* (2014)	W/S: 71%; L: 22%; P: 7%	ns	ns	33^‡^ (IQR: 42.5)	[73]
Renaud *et al.* (2014)	W: 79.5%; L: 18%; P: 2.5%	Th	ns	ns	[74]
Zampino *et al.* (2014)	W: 60.3%; S: 13.6%; L: 26.1%	ns	89.4%	48^‡^ (3.6–172.8)	[75]
Booth *et al.* (2015)	L: 40%; S: 56%; P: 4%	ns	ns	ns	[41]
Hou *et al.* (2015)	VATS: L: 33.3%: W: 66.7% Open: L: 36.8%; W: 63.2%	V: 50%; Th: 50%	VATS: 98.2% Open: 100%	45^‡^	[21]
Seebacher *et al.* (2015)	W/S/La: 94.5%; L: 5.5%	Th	ns	60	[76]
Guerrera *et al.* (2016)	W: 68.1%; S: 8.5%; L: 20.2%; P: 0.5%; C: 2.9%	Th: 80.9%; St: 18.1%; BTh: 1.0%	92%	45^‡^ (IQR: 30–69)	[77]
Filippi *et al.* (2016)	ns	ns	ns	ns	[30]
Franzke *et al.* (2016)	La: 55.6%; W: 34.8%; S: 5.1%; L: 4.5%	Th: 74.5%; V: 25.5%	99%	23.8^‡^ (2–66)	[78]
Girelli *et al.* (2016)	ns	ns	83.5%	52^‡^	[79]
Okiror *et al.* (2016)	ns	V: 73%; Th: 27%	ns	31^‡^ (1–60)	[80]
Guerrini *et al.* (2016)	L: 23%; W/S: 77%	V	ns	40^†^	[39]
Gafencu *et al.* (2017)	W/La: 85.1%; S: 4.9%; L:10.1%	U: 70.0%; Bi: 29.9%	ns	ns	[22]
Kim *et al.* (2017)	W: 81.4%; S: 8.5%; L: 7.0%; C: 3.1%	ns	ns	46.4^†^ (9–111)	[23]
Shiomi *et al.* (2017)	W: 76%; S/L: 24%	ns	100%	51	[81]
Sun *et al.* (2017)	W: 66.9%; S: 3.2%; L: 29.9%	V	ns	37^‡^ (2–138)	[82]
Shiono *et al.* (2017)	W: 82.3%; S: 17.7%	ns	100%	64.8^‡^	[14]
Lodeweges *et al.* (2017)	ns	ns	ns	7.6 years (5.8–9.8)	[24]

Follow-up values expressed in months as mean/median (range):

† Mean.

‡ Median.

An: Anatomical resection; B: Bilobectomy; Bi: Bilateral; Bio: Biopsy; BTh: Simultaneous bilateral thoracotomy; C: Combined resection; Cl: Clamshell; Eth: Exploratory thoracotomy; HAV: Substernal hand-assisted thoracoscopic approach; HCl: Hemiclamshell; IQR: Interquartile range; L: Lobectomy; La: Laser resection; ns: Not stated; O: Other; P: Pneumonectomy; R0: No residual disease; S: Segmentectomy; SL: sleeve lobectomy; St: Median sternotomy; STh: Staged thoracotomy; Th: Thoracotomy; TL: Thoracolaparotomy: Tu: Tumorectomy; U: Unilateral; V: Video-assisted thoracic surgery; W: Wedge resection.

There was wide heterogeneity in reporting follow-up time with 15 authors (24.6%) not mentioning it at all. Median follow-up was greater than 2 years in 67.2% and greater than 4 years in 18% of included articles.

#### SBRT

The description of treatments and length of follow-up is reported in Table 4. A wide range of treatment regimens were found within and across studies, in terms of prescribed doses of radiation and fractions of delivery. Several papers report percentage of isodose delivered at isocenter and PTV and biologically effective doses (BED_10_). All papers reported follow-up period, usually expressed as median and range. Median follow-up time was higher than 2 years in 44.4% of included articles, however, none of them reported a follow-up time of more than 4 years.

**Table 4.** Stereotactic radiotherapy: treatment and follow-up.

Study (year)	BED_10_	Total dose (Gy)	Fractions	Follow-up (mo)	Ref.
Hof *et al.* (2007)	ns	12–30	1	14^‡^ (1.5–82)	[6]
Ricardi *et al.* (2011)	ns	26–45	1–3	20.4^‡^ (3–77.4)	[83]
Zhang *et al.* (2011)	79.2–132	36–60	3–5	24.7^‡^ (2.9–114.4)	[25]
Oh *et al.* (2012)	100–150	50–60	4–5	21^‡^ (3–107)	[34]
Filippi *et al.* (2014)	94–138	26	1	24^‡^	[5]
Osti *et al.* (2013)	76–120	23–30	1	15^‡^ (3–45)	[29]
Siva *et al.* (2015)	ns	(18–50)	1–5	25^‡^	[84]
Aoki *et al.* (2015)	100 (85.5–120)	50 (45–60)	5 (5–9)	31.7^‡^ (4.3–130)	[85]
Binkley *et al.* (2015)	85 (50.4–151.2)	25 (18–50)	ns	22^‡^ (3–68)	[86]
Jung *et al.* (2015)	ns	40–60	3–4	42.8^‡^ (11.0–104.1)	[87]
De Rose *et al.* (2016)	ns	48–60	3–8	28.1^‡^ (5.4–104.5)	[88]
Kinj *et al.* (2016)	ns	50–75	3–5	33^‡^ (4–85)	[33]
Helou *et al.* (2016)	ns	48–52	4–5	22^‡^ (IQR 14–33)	[89]
Rieber *et al.* (2016)	84.4 (22.5–180)	12.5 (3–33)	3 (1–13)	14.3^‡^ (0–131.9)	[4]
Yamashita *et al.* (2016)	105.6 (75.0–134.4)	ns	ns	21^‡^ (1–119)	[90]
Franceschini *et al.* (2017)	ns	30–60	1–8	24.2^†^ (2.2–115.5)	[91]
Ricco *et al.* (2017)	100 (IQR: 81–136)	50 (IQR: 48–54)	3 (IQR: 3–5)	13^‡^ (IQR: 6–26)	[92]
Qiu *et al.* (2018)	ns	40–60	5–11	6.4^‡^	[93]

Values expressed as number (fractions) or median (range) or range:

† Mean.

‡ Median. Follow-up values expressed as months.

BED_10_: Biological effective dose; fr: Fraction; GTV: Gross tumor volume; Gy: Gray; IQR: Interquartile range; ns: Not stated; PTV: Planning tumor volume.

### Outcomes & Complications

#### Surgery

A description of the prognostic outcomes of surgical treatments and postoperative complications is reported in Table 5. Prognostic outcomes were variously described across studies: a survival analysis has been performed in the vast majority of articles, but intervals for assessing survivors’ prevalence ranged widely between short-term (1 and 2 years) and long-term lapses (5 and 10 years). Rates of survival at 1, 5 and 10 years ranged between 88–96%, 15–76% and 11–41%, respectively. Recurrence-free survival (RFS) and PFS were reported in a minor proportion of manuscripts (24.6%). PFS rates at 1 and 5 years were 45–87% and 5–64%, respectively. Postoperative mortality was reported in 73.8% of studies, ranging between 0 and 4.7%. In nearly half of the included studies, a clear mention of the prevalence of complications was not found or could not be derived from presented data. Where reported, overall complication rates ranged between 0 and 23%. A description or a list of such complications has been found in 59% of papers.

**Table 5.** Surgery: outcomes and complications.

Study (year)	OS	PFS	Median survival	Mortality	Morbidity	Ref.
Fourquier *et al.* (1997)	5 yrs: 44%	5 yrs; 25%	25	2%	6%	[12]
Robert *et al.* (1997)	2 yrs: 69%; 5 yrs: 48%; 10 yrs: 35%	ns	ns	1.8%	ns	[44]
Pastorino *et al.* (1997)	5 yrs: 36%; 10 yrs: 26%; 15 yrs: 22%	ns	35	1%	ns	[10]
Van der Veen *et al.* (1998)	5 yrs: 38%	ns	ns	0%	15.3%	[13]
Abecasis *et al.* (1999)	5 yrs: 29.2%	ns	ns	4.7%	21.2%	[45]
Kolodziejski *et al.* (1999)	5 yrs: 58%; 10 yrs: 38%	ns	58	2.1%	9.6%	[35]
Leo *et al.* (2000)	5 yrs: 22%; 10 yrs: 16%	ns	17	ns	ns	[46]
Friedel *et al.* (2001)	5 yrs: 35%; 10 yrs: 20%; 15 yrs: 18%	ns	35	ns	ns	[47]
Piltz *et al.* (2002)	ns	ns	61	0.95%	10.7%	[48]
Planchard *et al.* (2003)	3 yrs: 58%; 5 yrs: 45%; 10 yrs: 30%	1 yr: 87%; 3 yrs: 48%	50.4 (2–252)	0.8%	ns	[49]
Monteiro *et al.* (2004)	5 yrs: 47.4%; 10 yrs: 37.7%	5 yrs: 33.9%; 10 yrs: 19.1%	ns	0	11.5%	[36]
Vogelsang *et al.* (2004)	1 yr: 90%; 3 yrs: 47%	ns	ns	0%	3%	[50]
Shiono *et al.* (2005)	5 yrs: 61.4%	ns	ns	0%	ns	[51]
Casali *et al.* (2006)	5 yrs: 36%	5 yrs: 26%	ns	ns	ns	[52]
Furak *et al.* (2006)	5 yrs: 33.6%	ns	ns	0	ns	[53]
Harting *et al.* (2006)	3 yrs: 46.2%; 5 yrs: 29%	ns	ns	ns	11.8%	[54]
Rena *et al.* (2006)	5 yrs: 37%; 10 yrs: 15.8%	5 yrs: 28%; 10 yrs: 13%	ns	0.9%	7.9%	[55]
Assouad *et al.* (2007)	5 yrs: 34.4%	ns	35	0	15.4%	[15]
Melloni *et al.* (2007)	3 yrs: 50%; 5 yrs: 42%; 10 yrs: 30%	ns	37	ns	ns	[16]
Veronesi *et al.* (2007)	5 yrs: 60%	ns	ns	ns	ns	[56]
Dahabre *et al.* (2008)	5 yrs: 32.69%	ns	42	0	0	[9]
Smith *et al.* (2008)	5 yrs: 15%	5 yrs: 5%	16	3.7%	ns	[17]
Yoshimoto *et al.* (2008)	5 yrs: 54%; 10 yrs: 40%; 20 yrs: 25%	ns	75.6	1.1%	ns	[37]
Winter *et al.* (2008)	5 yrs: 20.9%	ns	19.4	4.4%	7.4%	[57]
Lin *et al.* (2009)	5 yrs: 43.9%; 10 yrs: 19.5%	5 yrs: 19.5%	ns	0%	0%	[58]
Rama *et al.* (2009)	3 yrs: 61%; 5 yrs: 48%; 10 yrs: 11%	ns	ns	0%	8%	[59]
Takakura *et al.* (2009)	3 yrs: 64.9%; 5 yrs: 48.2%	ns	ns	ns	ns	[60]
Borasio *et al.* (2010)	5 yrs: 55.4%; 10 yrs: 30.8%	ns	28.8 (6–99.3)	0	13.1%	[61]
Riquet *et al.* (2010)	5 yrs: 41%; 10 yrs: 27%	ns	45	0	14.5%	[62]
Sardenberg *et al.* (2010)	5 yrs: 34.7%	ns	36.7	0%	9.1%	[63]
Tacconi *et al.* (2010)	3 yrs: 57.9%; 5 yrs: 38.4%	3 yrs: 53.5%; 5 yrs: 40.9%	ns	0%	2.3%	[64]
Casiraghi *et al.* (2011)	2 yrs: 74%; 5 yrs: 46%	ns	ns	0	13.8%	[65]
Long *et al.* (2011)	3 yrs: 59.8%; 5 yrs: 47.2%	ns	40	0	3.6%	[66]
Stephens *et al.* (2011)	ns	ns	24.1	ns	ns	[67]
Zabaleta *et al.* (2011)	3 yrs: 70.2%; 5 yrs: 54.3%	ns	72 (0–129)	2%	8.3%	[68]
Blackmon *et al.* (2012)	5 yrs: 55.4%	ns	70.1	0	1.7%	[18]
Chua *et al.* (2012)	3 yrs: 41%; 5 yrs: 34%	1 yr: 45%; 3 yrs: 25%	23	1%	ns	[69]
Goonerante *et al.* (2012)	5 yrs: 40%	ns	ns	0%	14%	[19]
Hirosawa *et al.* (2012)	2 yrs: 76.6%; 5 yrs: 46.7%	ns	ns	0	ns	[70]
Mizuno *et al.* (2012)	ns	ns	50	ns	7.6%	[20]
Lo Faso *et al.* (2013)	ns	ns	ns	0	Major 2.4%	[71]
Poletti *et al.* (2013)	1 yr: 96%; 3 yrs: 77%; 5 yrs: 56%; 10 yrs: 39%	ns	ns	1.9%	22.1%	[38]
Cho *et al.* (2014)	5 yrs: 55.6%	5 yrs: 64.1%	ns	ns	ns	[72]
Renaud *et al.* (2014)	ns	ns	ns	2%	ns	[73]
Renaud *et al.* (2014)	1 yr: 88%; 2 yrs: 72%; 3 yrs: 66%; 5 yrs: 58%	ns	94	ns	ns	[74]
Zampino *et al.* (2014)	5 yrs: 43%	ns	50.4	0%	ns	[75]
Booth *et al.* (2015)	5 yrs: 40%; 10 yrs: 27%	ns	ns	0.9%	ns	[41]
Hou *et al.* (2015)	5 yrs: VATS: 50%; Open: 46%	ns	ns	0%	VATS: 19%; open: 23%	[21]
Seebacher *et al.* (2015)	5 yrs: 27.9%	ns	ns	0.3%	5.7%	[76]
Guerrera *et al.* (2016)	2 yrs: 80%; 5 yrs: 53%	2 yrs: 54%; 5 yrs: 33%	ns	0.5%	2%	[77]
Filippi *et al.* (2016)	1 yr: 96%; 2 yrs: 82%	ns	ns	1 (0.7%)	0%	[30]
Franzke *et al.* (2016)	1 yr: 88.2%; 3 yrs: 71.4%; 5 yrs: 69.3%	ns	51.0 (46.9–55.1)	0	11.5%	[78]
Girelli *et al.* (2016)	5 yrs: 66.8%; 10 yrs: 40.5%	ns	90.9	ns	ns	[79]
Okiror *et al.* (2016)	ns	ns	25.5 (1–60)	0%	ns	[80]
Guerrini *et al.* (2016)	1 yr: 88.2%; 3 yrs: 71.4%; 5 yrs: 60.6%; 10 yrs: 41.3%	ns	ns	0%	ns	[39]
Gafencu *et al.* (2017)	ns	ns	ns	0	2.75%	[22]
Kim *et al.* (2017)	5 yrs: 62.9%	3 yrs: 50.7%	ns	ns	ns	[23]
Shiomi *et al.* (2017)	5 yrs: 76%	5 yrs: 41%	ns	ns	ns	[81]
Sun *et al.* (2017)	5 yrs: 71.3%	ns	ns	ns	ns	[82]
Shiono *et al.* (2017)	5 yrs: 70.5%	5 yrs: 38.2%	ns	0%	6.5%	[14]
Lodeweges *et al.* (2017)	5 yrs: 41%	1 yr: 56%	ns	ns	ns	[24]

Survival values expressed as months (range).

ns: Not stated; OS: Overall survival; PFS: Progression-free survival; VATS: Video-assisted thoracic surgery.

#### SBRT

Outcomes of treatment were reported in an extensive manner in most papers (Table 6). The authors report rates of overall survival (OS) or RFS in almost all articles, with the majority of them (88.9%) mentioning percentages of LC, reported as rates of LC and progression, local failure-free survival or local response. Intervals for assessing survival and tumor progression were definitely shorter in SBRT papers: only five of the included articles (31.2%) reported percentages of LC and survival at 5 years. Rates of OS at 1, 2 and 5 years ranged between 74–94.5%, 31.2–74.6% and 21.8–58.3%, respectively. PFS at 1 and 2 years was 23.5–72% and 10–57%, respectively. Mortality rates have been reported in 83.3% of publications and in 72.2% of series no patients died as a consequence of ablative treatment. Post-treatment mortality ranged between 0 and 2%. Overall rates of adverse events were described in 27.8% of papers, whereas a detailed description of them, often with relative percentages or absolute numbers, could be encountered more frequently. Adverse events were almost always reported using the Common Terminology Criteria for Adverse Events [27] and their incidence ranged between 4 and 31%. In the majority of articles that reported adverse events were Grade 1–Grade 2 toxicity, without the need for further treatments or minor measures only.

**Table 6.** Stereotactic radiotherapy: outcomes and complications.

Study (year)	OS	PFS	LC	Mortality	Morbidity	Ref.
Hof *et al.* (2007)	1 yr: 78.4%; 2 yrs: 65.1%; 3 yrs: 47.8%	ns	1 yr: 88.6%; 2 yrs: 73.7%; 3 yrs: 63.1%	0%	ns	[6]
Ricardi *et al.* (2011)	2 yrs: 66.5%	2 yrs: 32.4%	ns	0%	4.92%	[83]
Zhang *et al.* (2011)	1 yr: 78.9%; 3 yrs: 40.8%; 5 yrs: 25.2%	ns	1 yr: 96.6%; 3 yrs: 89.4%; 5 yrs: 89.4%	0%	ns	[25]
Oh *et al.* (2012)	2 yrs: 59.7%; 5 yrs: 56.2%	ns	ns	2%	ns	[34]
Filippi *et al.* (2014)	1 yr: 85.1%; 2 yrs: 70.5%	1 yr: 72%; 2 yrs: 55.4%	1 yr: 93.4%; 2 yrs: 88.1%	0%	ns	[5]
Osti *et al.* (2013)	1 yr: 76.4%; 2 yrs: 31.2%	1 yr: 53.9%; 2 yrs: 22%	1 yr: 89.1%; 2 yrs: 82.1%	0%	ns	[29]
Siva *et al.* (2015)	1 yr: 93%; 2 yrs: 71%	ns	ns	0%	31%	[84]
Aoki *et al.* (2015)	3 yrs: 76%	3 yrs: 53.7%	3 yrs: 90%	0%	5%	[85]
Binkley *et al.* (2015)	1 yr: 93.7%; 2 yrs: 74.6%	ns	1 yr: 91.3%; 2 yr: 83.8%	0%	ns	[86]
Jung *et al.* (2015)	3 yrs: 64%	3 yrs: 24%	1 yr: 88.7%; 3 yrs: 70.6%	0%	4%	[87]
De Rose *et al.* (2016)	1 yr: 94.5%; 2 yrs: 74.6%; 3 yrs: 64.3%; 5 yrs: 22.1%	ns	ns	0%	ns	[88]
Kinj *et al.* (2016)	1 yr: 83.8%; 2 yrs: 69.3%; 5 yrs: 58.3%	1 yr: 29.2%; 2 yrs: 14.6%	1 yr: 79.8%; 2 yrs: 78.2%	0%	ns	[33]
Helou *et al.* (2016)	ns	ns	1 yr: 95.6%; 2 yrs: 84.8%	ns	8.3%	[89]
Rieber *et al.* (2016)	1 yr: 75.1%; 2 yrs: 54.4%	ns	1 yr: 90.9%; 2 yrs: 81.2%	0.2%	ns	[4]
Yamashita *et al.* (2016)	3 yrs: 53.2%	3 yrs: 32.2%	3 yrs: 74.2%	0%	ns	[90]
Franceschini *et al.* (2017)	ns	1 yr: 84%; 2 yrs: 57.7%; 3 yrs: 47%	1 yr: 91%; 2 yrs: 84.9%; 3 yrs: 82%	0%	ns	[91]
Ricco *et al.* (2017)	1 yr: 74.1%; 3 yrs: 33.3%; 5 yrs: 21.8%	ns	1 yr: 80.4%; 3 yrs: 58.9%; 5 yrs: 46.2%	ns	ns	[92]
Qiu *et al.* (2018)	1 yr: 77.8%; 2 yrs: 42.8%	1 yr: 23.5%; 2 yrs: 10.1%	1 yr: 56.6%; 2 yrs: 30.9%	ns	ns	[93]

LC: Local control; ns: Not stated; OS: Overall survival; PFS: Progression-free survival.

## Discussion

Surgical excision of pulmonary oligometastases seems to guarantee improved outcomes in terms of survival, and it is currently the first-choice treatment for this condition [28]. In contrast, SBRT has traditionally been reserved for patients unsuitable for surgical treatment. Nonetheless, recent improvements in technology and more precise protocols are broadening SBRT current indications [29]. It has been proposed that SBRT might induce not only tumor cell death, but also a tumor-specific response of the host immune system, inactivation of remnant micrometastasis and improved control of disease, in what is known as ‘abscopal effect’ [30]. Consequently, some authors evaluated SBRT as an effective and less invasive alternative to surgery for patients with lung oligometastases [31].

The aim of this study was to report the current evidence on these two approaches for treatment of pulmonary metastases and compare them in terms of effectiveness and safety.

The main findings of our study can be summarized as follows:

- We failed to demonstrate any substantial difference between surgery and SBRT in terms of short-term survival results.
- Data on long-term outcomes suggest an advantage for surgery in terms of survival.
- The incidence of adverse events was overall similar in the reported articles, although in the radiotherapy articles an overestimation of adverse events has been observed.

In the selected studies, OS at 1 year was 71–96% in surgical series and 74–94.5% after radiation therapy. A recent retrospective study comparing the two treatments for metastases from colorectal cancer demonstrated similar rates of survival in the short term, without significant differences in the two groups [32]. These figures are expression of a good outcome after resection/irradiation in the short term, but it is not clear whether this is a consequence of local ablative treatments or part of the natural history of the disease [33]. Patients with an oligometastatic state are a particular subset of subjects with favorable prognostic factors [33] and therefore, short-term survival is probably not the best parameter to establish the comparative effectiveness of these two treatments. A more appropriate indicator of therapeutic efficacy in the short term might be the PFS. In the present review, PFS was slightly higher in the surgical studies. In a recent retrospective study comparing surgery and SBRT for lung metastases, Filippi *et al.* demonstrated a worse outcome in terms of PFS and local progression among patients in the SBRT group [32]. Since in the context of metastatic disease the main goal of treatment is not cure but delaying progression and prolonging survival [34], the most important point is performing a procedure that eliminates any possible source of further metastatic spread. The presence of residual tumor has been demonstrated to be the main negative prognostic factor in many large surgical studies, some of them published in the late 90s [11]. While surgical excision with evaluation of resection margins allows an immediate feedback of completeness of resection, the efficacy of SBRT is defined with the concept of LC and it can only be assessed at follow-up investigations. The primary goal of SBRT for the treatment of oligometastases is to achieve an excellent rate of LC [31]. Although the R parameter refers to the degree of completeness of surgical resection and LC is a late measure of effectiveness of SBRT treatment, there is an evident analogy between the two factors in terms of potential eradication of disease. In the reported studies, slightly better rates of complete resection in the surgical series have been described when compared with LC at 1 year. This may have a significant impact on recurrence and PFS and therefore, on long-term prognosis.

Only a minority of reports on SBRT have a follow-up time long enough to describe long-term outcomes data, but from the included studies OS at 5 years tends to be higher in the surgical series. However, these results should be interpreted with caution, since SBRT is currently still reserved for patients unfit or unwilling to undergo a surgical treatment and this might have a considerable impact on the baseline conditions and survival rates of SBRT series populations. In SBRT studies the best outcomes seem to be reached with high doses of radiation: in the study from Kinj *et al.* [35]. 5-years OS reached 58.3% and in the series of Oh *et al.*, this was 56% [36]. This confirms that high doses of radiation conveyed to the target nodule, expressed as biological effective dose (BED_10_), result in better ablative power and control of disease [34]. However, the effectiveness of SBRT seems to depend not only on the irradiation protocols but also on the pattern of development of the oligometastatic state: in a recent large Japanese nationwide series, patients with a metachronous oligometastatic state demonstrated higher OS rates compared with those with synchronous oligometastasis [10]. Nonetheless, increasing attention is being paid toward the evolution of the pattern of disease in response to systemic therapy or previous local ablative treatments and future investigations will likely address the role of repeat-oligometastasis, the differences between a genuine or a chemotherapy-induced oligometastatic state and their impact on the prognosis of patients [37]. Moreover, the rapid evolution of systemic treatments and the introduction of molecular-targeted therapies might change the role of local aggressive treatments in this setting [38].

In the surgical series from Smith *et al.*, the authors report a low survival rate of 15% at 5 years and in the same study, complete resection resulted the most relevant factor affecting survival [18]. In this study the prevalence of R0 resections was low (78%), confirming the importance of a complete eradication of tumor deposits during surgical treatment. Apart from this exceptions, 5-years OS is usually greater in surgical studies and can be as high as 76%. Moreover, several surgical series describe rates of survival at 10 years reaching 40% [39–43], suggesting that a stabilization or eradication of disease can be obtained in some cases.

Complications and adverse events were reported with similar percentages between surgical and SBRT series, but this is probably the result of a reporting bias: there is an overestimation of adverse events after SBRT, due to reporting of complications that do not require any further therapeutic measure.

There are consistent differences between surgical excision of metastases and other ablative techniques: excision of lung nodules allows availability of tissue for confirmation of the metastatic nature of nodules, assessment of resection margin for complete resection and pathological study for existing or future targeted therapies [44].

SBRT is an ablative technique that does not allow tissue harvesting for confirmation of diagnosis. Thus, one may wonder whether all sites of irradiation effectively correspond to metastatic deposits. Indication for irradiation is usually given at multidisciplinary tumor boards, based on patients' past medical history, radiological features of the nodules and dimensional evolution. Nevertheless, even with high levels of suspicion, some benign nodules may mimic tumor features in radiological examinations. Indeed, several surgical investigations report how some excised nodules did not confirm their metastatic nature at histopathological analysis [45,46]. Therefore, including patients with benign nodules treated with SBRT might overestimate the survival benefits in the whole population.

Another element of concern could be that treating patients with SBRT means not having any information regarding the R0 status after treatment or possible nodal involvement. Moreover, a suboptimal sensitivity of preoperative imaging techniques (CT, CT-PET) has been demonstrated [47] and unexpected further lung nodules at thoracotomy are not an uncommon finding [46,47]. For SBRT, this might implicate that radiation is not delivered on a potential source of further tumor dissemination.

Finally, our investigation demonstrated that surgical series provide information based on larger populations and with longer follow-up times. This makes, in our opinion, results coming from surgical reports more reliable. As long as greater experience will mature in the use of SBRT and more solid evidence will emerge from large series, we still recommend surgery as the main therapeutic option for oligometastatic disease of the lung. A prospective randomized protocol comparing the two techniques is required to confirm our conclusions.

## Limitations

There are several limitations in this study. The low number of articles directly comparing the two therapeutic options lead the authors to include series dealing with one of the two treatments only. Nonetheless, most of the articles reported incomplete information on outcomes and baseline conditions of patients and this may have reduced the reliability of results. The net prevalence of retrospective studies and the presence of bias within and across them may have limited the accuracy of reported evidence. Including studies with metastases from several primary tumors may have limited the significance of the outcome results, but this was determined by the fact that most SBRT series deal with metastases arising from multiple histology. SBRT is usually reserved to patients excluded from surgical indication due to anatomical considerations or general conditions: this may have an impact on prognostic outcomes. SBRT articles described adverse events in a more accurate manner, reporting even low-grade events where there was no need for any intervention: this undoubtedly overestimated the overall reported incidence of complications compared with surgical series. Nonetheless many surgical articles did not report the R category, which is an aspect of main importance when dealing with a therapeutic resection for a neoplastic state.

## Conclusion

Surgical resection of pulmonary oligometastases is a safe and effective practice and allows a good control of disease and prolongation of life, in cases where a complete resection can be achieved. SBRT is an attractive option with lower invasiveness and side effects, but long-term follow-up data are still limited. Nonetheless, the heterogeneity of therapy protocols in SBRT and the intrinsic differences between the two treatments do not allow to draw a conclusion on the superiority of one option over the other.

## Future perspective

The recent technological evolution led to a better understanding of the tumors’ behavior and the introduction of new molecules changed the view of metastatic disease from being a condition of negative outcome to a stage where the tumor can be stabilized or eradicated. This draws interesting perspectives:

- What will be the role of local treatments in the context of an ‘induced oligometastatic state’ after systemic therapy regimens administered on patients with an overt polimetastatic disease?
- In this setting, it would be of great interest to assess the new roles of the different ablative techniques during the evolution of disease;
- Nonetheless, a Phase III trial with long follow-up times and strict inclusion criteria would be recommended to compare the effectiveness of the different aggressive local treatments in the context of oligometastatic disease.

**Executive summary**

- An oligometastatic state is a condition with a limited number of metastases, where local aggressive treatments, such as surgical resection or stereotactic radiotherapy (SBRT), may play an important therapeutic role.
- Aim of the study was to define which is the best local treatment for patients with an oligometastatic disease.
- The study was conducted according to the PRISMA guidelines for systematic review and meta-analysis and literature search was performed by defining a searching strategy using the PICOS method.
- Only articles in English language, with populations of more than 50 patients and where the treatment was performed with curative intent were included.
- Sixty-one surgical studies and 18 articles on SBRT were finally selected.
- Overall survival is comparable in the short term but tends to be higher in the long term, although there is a scarcity of long-term results for SBRT.
- Progression-free survival at 1 and 2 years tends to be higher in surgical studies.
- Mortality is comparable between surgery and SBRT, while the incidence of significant adverse events is higher in patients undergoing surgical resection.
- The intrinsic differences between the two techniques and the heterogeneity of reporting of the included articles do not consent to define which is the best treatment.
- Future investigations should include a prospective trial to compare the two modalities of treatment and define the role of the different ablative techniques in the era of new chemotherapy regimens.

## References

[1] Gloeckler Ries LA, Reichman ME, Lewis DR, Hankey BF, Edwards BK Cancer survival and incidence from the Surveillance, Epidemiology, and End Results (SEER) program. Oncologist 8(6), 541–552 (2003). 14657533 10.1634/theoncologist.8-6-541

[2] Sauter ER, Bolton JS, Willis GW, Farr GH, Sardi A Improved survival after pulmonary resection of metastatic colorectal carcinoma. J. Surg. Oncol. 43(3), 135–138 (1990). 2314100 10.1002/jso.2930430303

[3] Timmerman RD, Bizekis CS, Pass HI Local surgical, ablative, and radiation treatment of metastases. CA Cancer J. Clin. 59(3), 145–170 (2009). 19364702 10.3322/caac.20013

[4] Rieber J, Streblow J, Uhlmann L Stereotactic body radiotherapy (SBRT) for medically inoperable lung metastases-a pooled analysis of the German working group “stereotactic radiotherapy”. Lung Cancer 97, 51–58 (2016). 27237028 10.1016/j.lungcan.2016.04.012

[5] Filippi AR, Badellino S, Guarneri A Outcomes of single fraction stereotactic ablative radiotherapy for lung metastases. Technol. Cancer Res. Treat 13(1), 37–45 (2014). 23819496 10.7785/tcrt.2012.500355

[6] Hof H, Hoess A, Oetzel D, Debus J, Herfarth K Stereotactic single-dose radiotherapy of lung metastases. Strahlenther Onkol. 183(12), 673–678 (2007). 18040611 10.1007/s00066-007-1724-z

[7] Moher D, Liberati A, Tetzlaff J, Altman DG Preferred reporting items for systematic reviews and meta-analyses: the PRISMA statement. BMJ (Clin. Rese. Ed.) 339, b2535 (2009). 10.1136/bmj.b2535 PMC2714657 19622551

[8] O'Connor DGS, Higgins JPT Chapter 5: defining the review question and developing criteria for including studies. In: Cochrane Handbook for Systematic Reviews and Interventions Version 5.1.0. (2008).

[9] Dahabre J, Vasilaki M, Stathopoulos GP Surgical management in lung metastases from colorectal cancer. Anticancer Res. 27(6c), 4387–4390 (2007). 18214049

[10] Niibe Y, Yamamoto T, Onishi H Pulmonary oligometastases treated by stereotactic body radiation therapy: a nationwide survey of 1,378 patients. Anticancer Res. 40(1), 393–399 (2020). 31892592 10.21873/anticanres.13965

[11] Pastorino U, Buyse M, Friedel G Long-term results of lung metastasectomy: prognostic analyses based on 5206 cases. J. Thorac. Cardiovasc. Surg. 113(1), 37–49 (1997). 9011700 10.1016/s0022-5223(97)70397-0

[12] Verazin GT, Warneke JA, Driscoll DL, Karakousis C, Petrelli NJ, Takita H Resection of lung metastases from soft-tissue sarcomas. A multivariate analysis. Arch. Surg. 127(12), 1407–1411 (1992). 1365685 10.1001/archsurg.1992.01420120041007

[13] Fourquier P, Regnard JF, Rea S, Levi JF, Levasseur P Lung metastases of renal cell carcinoma: results of surgical resection. Eur. J. Cardiothorac. Surg. 11(1), 17–21 (1997). 9030784 10.1016/s1010-7940(96)01013-5

[14] van der Veen AH, van Geel AN, Hop WC, Wiggers T Median sternotomy: the preferred incision for resection of lung metastases. Eur. J. Surg. 164(7), 507–512 (1998). 9696972 10.1080/110241598750005859

[15] Shiono S, Ishii G, Nagai K Histopathologic prognostic factors in resected colorectal lung metastases. Ann. Thorac. Surg. 79(1), 278–282 (2005). 15620957 10.1016/j.athoracsur.2004.06.096

[16] Assouad J, Petkova B, Berna P, Dujon A, Foucault C, Riquet M Renal cell carcinoma lung metastases surgery: pathologic findings and prognostic factors. Ann. Thorac. Surg. 84(4), 1114–1120 (2007). 17888956 10.1016/j.athoracsur.2007.04.118

[17] Melloni G, Doglioni C, Bandiera A Prognostic factors and analysis of microsatellite instability in resected pulmonary metastases from colorectal carcinoma. Ann. Thorac. Surg. 81(6), 2008–2013 (2006). 16731121 10.1016/j.athoracsur.2006.01.007

[18] Smith R, Pak Y, Kraybill W, Kane JM 3rd Factors associated with actual long-term survival following soft tissue sarcoma pulmonary metastasectomy. Eur. J. Surg. Oncol. 35(4), 356–361 (2009). 18294807 10.1016/j.ejso.2008.01.004

[19] Blackmon SH, Stephens EH, Correa AM Predictors of recurrent pulmonary metastases and survival after pulmonary metastasectomy for colorectal cancer. Ann. Thorac. Surg. 94(6), 1802–1809 (2012). 23063195 10.1016/j.athoracsur.2012.07.014

[20] Goonerante D, Gray C, Lim M Survival outcome in New Zealand after resection of colorectal cancer lung metastases. ANZ. J. Surg 83(12), 959–962 (2013). 23186081 10.1111/ans.12012

[21] Mizuno T, Taniguchi T, Ishikawa Y Pulmonary metastasectomy for osteogenic and soft tissue sarcoma: who really benefits from surgical treatment? Eur. J. Cardiothorac. Surg. 43(4), 795–799 (2013). 22833540 10.1093/ejcts/ezs419

[22] Hou Z, Zhang H, Gui L, Wang W, Zhao S Video-assisted thoracoscopic surgery versus open resection of lung metastases from colorectal cancer. Int. J. Clin. Exp. Med. 8(8), 13571–13577 (2015). 26550296 PMC4612981

[23] Gafencu DA, Welter S, Cheufou DH Pulmonary metastasectomy for sarcoma-Essen experience. J. Thorac. Dis 9(Suppl. 12), S1278–S1281 (2017). 29119015 10.21037/jtd.2017.07.47 PMC5653502

[24] Kim JY, Park IJ, Kim HR Post-pulmonary metastasectomy prognosis after curative resection for colorectal cancer. Oncotarget 8(22), 36566–36577 (2017). 28402263 10.18632/oncotarget.16616 PMC5482677

[25] Lodeweges JE, Klinkenberg TJ, Ubbels JF, Groen HJM, Langendijk JA, Widder J Long-term outcome of surgery or stereotactic radiotherapy for lung oligometastases. J. Thorac. Oncol 12(9), 1442–1445 (2017). 28576747 10.1016/j.jtho.2017.05.015

[26] Zhang Y, Xiao JP, Zhang HZ Stereotactic body radiation therapy favors long-term overall survival in patients with lung metastases: five-year experience of a single-institution. Chin. Med. J. 124(24), 4132–4137 (2011). 22340374

[27] Trotti A, Colevas AD, Setser A CTCAE v3.0: development of a comprehensive grading system for the adverse effects of cancer treatment. Semin. Radiat. Oncol. 13(3), 176–181 (2003). 12903007 10.1016/S1053-4296(03)00031-6

[28] Navarria P, Ascolese AM, Cozzi L Stereotactic body radiation therapy for lung metastases from soft tissue sarcoma. Eur. J. Cancer 51(5), 668–674 (2015). 25686482 10.1016/j.ejca.2015.01.061

[29] DeLaney TF, Trofimov AV, Engelsman M, Suit HD Advanced-technology radiation therapy in the management of bone and soft tissue sarcomas. Cancer Control. 12(1), 27–35 (2005). 15668650 10.1177/107327480501200104

[30] Niibe Y, Chang JY Novel insights of oligometastases and oligo-recurrence and review of the literature. Pulm. Med. 2012, 261096–261096 (2012). 22966429 10.1155/2012/261096 PMC3432385

[31] Osti MF, Carnevale A, Valeriani M Clinical outcomes of single dose stereotactic radiotherapy for lung metastases. Clin. Lung Cancer 14(6), 699–703 (2013). 23886798 10.1016/j.cllc.2013.06.006

[32] Filippi AR, Guerrera F, Badellino S Exploratory analysis on overall survival after either surgery or stereotactic radiotherapy for lung oligometastases from colorectal cancer. Clin. Oncol. 28(8), 505–512 (2016). 10.1016/j.clon.2016.02.001 26899780

[33] Treasure T, Milosevic M, Fiorentino F, Macbeth F Pulmonary metastasectomy: what is the practice and where is the evidence for effectiveness? Thorax 69(10), 946–949 (2014). 24415715 10.1136/thoraxjnl-2013-204528 PMC4174129

[34] Tree AC, Khoo VS, Eeles RA Stereotactic body radiotherapy for oligometastases. Lancet Oncol. 14(1), e28–e37 (2013). 23276369 10.1016/S1470-2045(12)70510-7

[35] Kinj R, Bondiau PY, Francois E Radiosensitivity of colon and rectal lung oligometastasis treated with stereotactic ablative radiotherapy. Clin. Colorectal Cancer 16(3), e211–e220 (2017). 27670890 10.1016/j.clcc.2016.08.003

[36] Oh D, Ahn YC, Seo JM Potentially curative stereotactic body radiation therapy (SBRT) for single or oligometastasis to the lung. Acta. Oncol. 51(5), 596–602 (2012). 22548366 10.3109/0284186X.2012.681698

[37] Guckenberger M, Lievens Y, Bouma AB Characterisation and classification of oligometastatic disease: a European Society for Radiotherapy and Oncology and European Organisation for Research and Treatment of Cancer consensus recommendation. Lancet Oncol. 21(1), e18–e28 (2020). 31908301 10.1016/S1470-2045(19)30718-1

[38] Niibe Y, Hayakawa K Oligometastases and oligo-recurrence: the new era of cancer therapy. Jpn. J. Clin. Oncol. 40(2), 107–111 (2010). 20047860 10.1093/jjco/hyp167 PMC2813545

[39] Koodziejski L, Goralczyk J, Dyczek S, Duda K, Nabiaek T The role of surgery in lung metastases. Eur. J. Surg. Oncol. 25(4), 410–417 (1999). 10421563 10.1053/ejso.1999.0667

[40] Monteiro A, Arce N, Bernardo J, Eugenio L, Antunes MJ Surgical resection of lung metastases from epithelial tumors. Ann. Thorac. Surg. 77(2), 431–437 (2004). 14759411 10.1016/j.athoracsur.2003.06.012

[41] Yoshimoto M, Tada K, Nishimura S Favourable long-term results after surgical removal of lung metastases of breast cancer. Breast Cancer Res. Treat. 110(3), 485–491 (2008). 17899365 10.1007/s10549-007-9747-9

[42] Poletti GB, Toro IF, Alves TF, Miranda EC, Seabra JC, Mussi RK Descriptive analysis of and overall survival after surgical treatment of lung metastases. J. Bras. Pneumol. 39(6), 650–658 (2013). 24473758 10.1590/S1806-37132013000600003 PMC4075905

[43] Guerrini GP, Lo Faso F, Vagliasindi A The role of minimally invasive surgery in the treatment of lung metastases. J. Invest. Surg. 30(2), 110–115 (2017). 27690700 10.1080/08941939.2016.1230246

[44] Treasure T Surgery and ablative techniques for lung metastases in the Pulmonary Metastasectomy in Colorectal Cancer (PulMiCC) trial: is there equivalence? J. Thorac. Dis. 8(Suppl. 9), S649–S651 (2016). 28066664 10.21037/jtd.2016.06.50 PMC5179353

[45] Booth CM, Nanji S, Wei X, Mackillop WJ Outcomes of resected colorectal cancer lung metastases in routine clinical practice: a population-based study. Ann. Surg. Oncol. 23(4), 1057–1063 (2016). 10.1245/s10434-015-4979-0 26572752

[46] Eckardt J, Licht PB Thoracoscopic or open surgery for pulmonary metastasectomy: an observer blinded study. Ann. Thorac. Surg. 98(2), 466–469 (2014). 24928676 10.1016/j.athoracsur.2014.04.063

[47] Guerrera F, Renaud S, Schaeffer M Low accuracy of computed tomography and positron emission tomography to detect lung and lymph node metastases of colorectal cancer. Ann. Thorac. Surg. 104(4), 1194–1199 (2017). 28760464 10.1016/j.athoracsur.2017.05.002

[48] Robert JH, Ambrogi V, Mermillod B, Dahabreh D, Goldstraw P Factors influencing long-term survival after lung metastasectomy. Ann. Thorac. Surg. 63(3), 777–784 (1997). 9066401 10.1016/s0003-4975(96)01103-4

[49] Abecasis N, Cortez F, Bettencourt A, Costa CS, Orvalho F, de Almeida JM Surgical treatment of lung metastases: prognostic factors for long-term survival. J. Surg. Oncol. 72(4), 193–198 (1999). 10589033 10.1002/(sici)1096-9098(199912)72:4<193::aid-jso3>3.0.co;2-8

[50] Leo F, Cagini L, Rocmans P Lung metastases from melanoma: when is surgical treatment warranted? Br. J. Cancer 83(5), 569–572 (2000). 10944593 10.1054/bjoc.2000.1335 PMC2363508

[51] Friedel G, Pastorino U, Ginsberg RJ Results of lung metastasectomy from breast cancer: prognostic criteria on the basis of 467 cases of the International Registry of Lung Metastases. Eur. J. Cardiothorac. Surg. 22(3), 335–344 (2002). 12204720 10.1016/s1010-7940(02)00331-7

[52] Piltz S, Meimarakis G, Wichmann MW, Hatz R, Schildberg FW, Fuerst H Long-term results after pulmonary resection of renal cell carcinoma metastases. Ann. Thorac. Surg. 73(4), 1082–1087 (2002). 11996245 10.1016/s0003-4975(01)03602-5

[53] Planchard D, Soria JC, Michiels S Uncertain benefit from surgery in patients with lung metastases from breast carcinoma. Cancer 100(1), 28–35 (2004). 14692021 10.1002/cncr.11881

[54] Vogelsang H, Haas S, Hierholzer C, Berger U, Siewert JR, Prauer H Factors influencing survival after resection of pulmonary metastases from colorectal cancer. Br. J. Surg. 91(8), 1066–1071 (2004). 15286972 10.1002/bjs.4602

[55] Shiono S, Okumura T, Boku N Outcomes of segmentectomy and wedge resection for pulmonary metastases from colorectal cancer. Eur. J. Cardiothorac. Surg. 51(3), 504–510 (2017). 27773868 10.1093/ejcts/ezw322

[56] Casali C, Stefani A, Storelli E, Morandi U Prognostic factors and survival after resection of lung metastases from epithelial tumours. Interact. Cardiovasc. Thorac. Surg. 5(3), 317–321 (2006). 17670578 10.1510/icvts.2005.125856

[57] Furak J, Trojan I, Szoke T, Tiszlavicz L, Eller J, Lazar G Visceral pleural infiltration as a negative prognostic factor in lung metastasis. Interact. Cardiovasc. Thorac. Surg. 6(2), 196–199 (2006). 10.1510/icvts.2006.143578 17669808

[58] Harting MT, Blakely ML, Jaffe N Long-term survival after aggressive resection of pulmonary metastases among children and adolescents with osteosarcoma. J. Pediatr. Surg. 41(1), 194–199 (2006). 16410132 10.1016/j.jpedsurg.2005.10.089

[59] Rena O, Papalia E, Oliaro A Pulmonary metastases from epithelial tumours: late results of surgical treatment. Eur. J. Cardiothorac. Surg. 30(2), 217–222 (2006). 16828294 10.1016/j.ejcts.2006.04.032

[60] Veronesi G, Petrella F, Leo F Prognostic role of lymph node involvement in lung metastasectomy. J. Thorac. Cardiovasc. Surg. 133(4), 967–972 (2007). 17382635 10.1016/j.jtcvs.2006.09.104

[61] Winter H, Meimarakis G, Hoffmann G Does surgical resection of pulmonary metastases of head and neck cancer improve survival? Ann. Surg. Oncol. 15(10), 2915–2926 (2008). 18648883 10.1245/s10434-008-0001-4

[62] Lin BR, Chang TC, Lee YC, Lee PH, Chang KJ, Liang JT Pulmonary resection for colorectal cancer metastases: duration between cancer onset and lung metastasis as an important prognostic factor. Ann. Surg. Oncol. 16(4), 1026–1032 (2009). 19184237 10.1245/s10434-008-0286-3

[63] Rama N, Monteiro A, Bernardo JE, Eugenio L, Antunes MJ Lung metastases from colorectal cancer: surgical resection and prognostic factors. Eur. J. Cardiothorac. Surg. 35(3), 444–449 (2009). 19136273 10.1016/j.ejcts.2008.10.047

[64] Takakura Y, Miyata Y, Okajima M, Okada M, Ohdan H Short disease-free interval is a significant risk factor for intrapulmonary recurrence after resection of pulmonary metastases in colorectal cancer. Colorectal. Dis. 12(7 Online), e68–e75 (2010). 19843115 10.1111/j.1463-1318.2009.02070.x

[65] Borasio P, Gisabella M, Bille A Role of surgical resection in colorectal lung metastases: analysis of 137 patients. Int. J. Colorectal. Dis. 26(2), 183–190 (2011). 20960207 10.1007/s00384-010-1075-6

[66] Riquet M, Foucault C, Cazes A Pulmonary resection for metastases of colorectal adenocarcinoma. Ann. Thorac. Surg. 89(2), 375–380 (2010). 20103301 10.1016/j.athoracsur.2009.10.005

[67] Sardenberg RAdS, Figueiredo LPd, Haddad FJ, Gross JL, Younes RN Pulmonary metastasectomy from soft tissue sarcomas. Clinics 65(9), 871–876 (2010). 21049215 10.1590/S1807-59322010000900010 PMC2954738

[68] Tacconi F, Ambrogi V, Pompeo E, Sellitri F, Mineo TC Substernal hand-assisted videothoracoscopic lung metastasectomy: long term results in a selected patient cohort. Thor. Cancer 2(2), 45–53 (2011). 10.1111/j.1759-7714.2010.00038.x 27755812

[69] Casiraghi M, De Pas T, Maisonneuve P A 10-year single-center experience on 708 lung metastasectomies: the evidence of the “international registry of lung metastases”. J. Thor. Oncol. 6(8), 1373–1378 (2011). 10.1097/JTO.0b013e3182208e58 21642869

[70] Long H, Zheng Y, Situ D, Ma G, Lin Z, Wang J Hand-assisted thoracoscopic surgery for bilateral lung metastasectomy through sternocostal triangle access. Ann. Thorac. Surg. 91(3), 852–858 (2011). 21353013 10.1016/j.athoracsur.2010.11.057

[71] Stephens EH, Blackmon SH, Correa AM Progression after chemotherapy is a novel predictor of poor outcomes after pulmonary metastasectomy in sarcoma patients. J. Am. Coll. Surg. 212(5), 821–826 (2011). 21435923 10.1016/j.jamcollsurg.2011.01.007

[72] Zabaleta J, Aguinagalde B, Fuentes MG Survival after lung metastasectomy for colorectal cancer: importance of previous liver metastasis as a prognostic factor. Eur. J. Surg. Oncol. 37(9), 786–790 (2011). 21723689 10.1016/j.ejso.2011.05.014

[73] Chua TC, Scolyer RA, Kennedy CW, Yan TD, McCaughan BC, Thompson JF Surgical management of melanoma lung metastasis: an analysis of survival outcomes in 292 consecutive patients. Ann. Surg. Oncol. 19(6), 1774–1781 (2012). 22290565 10.1245/s10434-011-2197-y

[74] Hirosawa T, Itabashi M, Ohnuki T Prognostic factors in patients undergoing complete resection of pulmonary metastases of colorectal cancer: a multi-institutional cumulative follow-up study. Surg. Today 43(5), 494–499 (2013). 23085967 10.1007/s00595-012-0373-8

[75] Lo Faso F, Solaini L, Lembo R Thoracoscopic lung metastasectomies: a 10-year, single-center experience. Surg. Endosc. 27(6), 1938–1944 (2013). 23344503 10.1007/s00464-012-2691-8 PMC3661047

[76] Cho JH, Hamaji M, Allen MS The prognosis of pulmonary metastasectomy depends on the location of the primary colorectal cancer. Ann. Thorac. Surg. 98(4), 1231–1237 (2014). 25086943 10.1016/j.athoracsur.2014.05.023

[77] Renaud S, Alifano M, Falcoz PE Does nodal status influence survival? Results of a 19-year systematic lymphadenectomy experience during lung metastasectomy of colorectal cancer. Interact. Cardiovasc. Thorac. Surg. 18(4), 482–487 (2014). 24442624 10.1093/icvts/ivt554 PMC3957298

[78] Renaud S, Falcoz PE, Alifano M Systematic lymph node dissection in lung metastasectomy of renal cell carcinoma: an 18 years of experience. J. Surg. Oncol. 109(8), 823–829 (2014). 24619772 10.1002/jso.23593

[79] Zampino MG, Maisonneuve P, Ravenda PS Lung metastases from colorectal cancer: analysis of prognostic factors in a single institution study. Ann. Thorac. Surg. 98(4), 1238–1245 (2014). 25106681 10.1016/j.athoracsur.2014.05.048

[80] Seebacher G, Decker S, Fischer JR, Held M, Schafers HJ, Graeter TP Unexpected lymph node disease in resections for pulmonary metastases. Ann. Thorac. Surg. 99(1), 231–236 (2015). 25440271 10.1016/j.athoracsur.2014.08.038

[81] Guerrera F, Mossetti C, Ceccarelli M Surgery of colorectal cancer lung metastases: analysis of survival, recurrence and re-surgery. J. Thorac. Dis. 8(7), 1764–1771 (2016). 27499967 10.21037/jtd.2016.05.98 PMC4958892

[82] Franzke K, Natanov R, Zinne N Pulmonary metastasectomy - a retrospective comparison of surgical outcomes after laser-assisted and conventional resection. Eur. J. Surg. Oncol. 43(7), 1357–1364 (2017). 27771210 10.1016/j.ejso.2016.09.001

[83] Girelli L, Locati L, Galeone C Lung metastasectomy in adenoid cystic cancer: is it worth it? Oral. Oncol. 65, 114–118 (2017). 28341276 10.1016/j.oraloncology.2016.10.018

[84] Okiror L, Peleki A, Moffat D Survival following pulmonary metastasectomy for sarcoma. Thorac. Cardiovasc. Surg. 64(2), 146–149 (2016). 25742552 10.1055/s-0035-1546430

[85] Shiomi K, Naito M, Sato T Effect of adjuvant chemotherapy after pulmonary metastasectomy on the prognosis of colorectal cancer. Ann. Med. Surg. (Lond.) 20, 19–25 (2017). 28702182 10.1016/j.amsu.2017.06.026 PMC5484968

[86] Sun F, Chen L, Shi M Prognosis of video-assisted thoracoscopic pulmonary metastasectomy in patients with colorectal cancer lung metastases: an analysis of 154 cases. Int. J. Colorectal Dis. 32(6), 897–905 (2017). 28176005 10.1007/s00384-017-2768-x

[87] Ricardi U, Filippi AR, Guarneri A Stereotactic body radiation therapy for lung metastases. Lung Cancer 75(1), 77–81 (2012). 21726918 10.1016/j.lungcan.2011.04.021

[88] Siva S, Kirby K, Caine H Comparison of single-fraction and multi-fraction stereotactic radiotherapy for patients with 18F-fluorodeoxyglucose positron emission tomography-staged pulmonary oligometastases. Clin. Oncol. 27(6), 353–361 (2015). 10.1016/j.clon.2015.01.004 25698068

[89] Aoki M, Hatayama Y, Kawaguchi H Stereotactic body radiotherapy for lung metastases as oligo-recurrence: a single institutional study. J. Radiat. Res. 57(1), 55–61 (2016). 26494115 10.1093/jrr/rrv063 PMC4708917

[90] Binkley MS, Trakul N, Jacobs LR Colorectal histology is associated with an increased risk of local failure in lung metastases treated with stereotactic ablative radiation therapy. Int. J. Radiat. Oncol. Biol. Phys. 92(5), 1044–1052 (2015). 26025776 10.1016/j.ijrobp.2015.04.004

[91] Jung J, Song SY, Kim JH Clinical efficacy of stereotactic ablative radiotherapy for lung metastases arising from colorectal cancer. Radiat. Oncol. 10, 238 (2015). 26588896 10.1186/s13014-015-0546-x PMC4654895

[92] De Rose F, Cozzi L, Navarria P Clinical outcome of stereotactic ablative body radiotherapy for lung metastatic lesions in non-small cell lung cancer oligometastatic patients. Clin. Oncol. 28(1), 13–20 (2016). 10.1016/j.clon.2015.08.011 26385822

[93] Helou J, Thibault I, Poon I Stereotactic ablative radiation therapy for pulmonary metastases: histology, dose, and indication matter. Int. J. Radiat. Oncol. Biol. Phys. 98(2), 419–427 (2017). 28463162 10.1016/j.ijrobp.2017.02.093

[94] Yamashita H, Niibe Y, Yamamoto T Lung stereotactic radiotherapy for oligometastases: comparison of oligo-recurrence and sync-oligometastases. Jpn. J. Clin. Oncol. 46(7), 687–691 (2016). 27162324 10.1093/jjco/hyw047 PMC4957009

[95] Franceschini D, Cozzi L, De Rose F Role of stereotactic body radiation therapy for lung metastases from radio-resistant primary tumours. J. Cancer Res. Clin. Oncol. 143(7), 1293–1299 (2017). 28258344 10.1007/s00432-017-2373-y PMC11819141

[96] Ricco A, Davis J, Rate W Lung metastases treated with stereotactic body radiotherapy: the RSSearch(R) patient Registry's experience. Radiat. Oncol. 12(1), 35 (2017). 28143558 10.1186/s13014-017-0773-4 PMC5286804

[97] Qiu H, Katz AW, Chowdhry AK Stereotactic body radiotherapy for lung metastases from colorectal cancer: prognostic factors for disease control and survival. Am. J. Clin. Oncol. 41(1), 53–58 (2018). 26270442 10.1097/COC.0000000000000220

